# Daily associations between peer victimization and anxious affect among adolescents: The role of social threat sensitivity

**DOI:** 10.1017/S0954579425100394

**Published:** 2025-08-06

**Authors:** Hannah L. Schacter, Hilary A. Marusak, Leah Gowatch, Tanja Jovanovic

**Affiliations:** 1 Department of Psychology, Wayne State University, Detroit, MI, USA; 2 Department of Psychiatry and Behavioral Neurosciences, Wayne State University, Detroit, MI, USA

**Keywords:** Adolescence, anxiety, daily diary, peer victimization, threat sensitivity

## Abstract

Adolescents frequently victimized by peers are two to three times more likely to develop an anxiety disorder than their non-victimized peers. However, the fine-grained mechanisms that explain how peer victimization confers risk for anxiety in adolescents’ daily lives are not well-understood. Leveraging an intensive longitudinal design, this study examined same- and cross-day links between peer victimization and anxiety, investigating social threat sensitivity as a potential underlying mechanism. One hundred ninety-five adolescents (*M*_age_ = 16.48, *SD*_age_ = 0.35; 66% female, 27% male, 11% non-binary, identifying with another gender; 48% White, 20% Asian, 15% Black, 17% identifying with another race/ethnicity) completed brief daily assessments of peer victimization, social threat sensitivity, and anxious affect for 14 days. Multilevel analyses indicated that adolescents reported greater anxious affect on days when they experienced peer victimization compared to days without victimization. Although peer victimization did not predict anxious affect the following day, it was associated with increased anxious affect two days later. Social threat sensitivity significantly mediated the same-day, but not cross-day, association between peer victimization and anxious affect, controlling for prior-day threat sensitivity and anxiety. The findings suggest that heightened social vigilance partially accounts for anxious affect in adolescents facing peer victimization in daily life.

## Introduction

Approximately one in every three adolescents has been victimized by their peers at some point in time, with nearly 20% enduring chronic harassment over multiple weeks or months (United Nations Educational, Scientific and Cultural Organization, 2019). Peer victimization places youth at heightened risk for mental health difficulties including anxiety (Christina et al., 2021), which is the most common pediatric psychiatric condition (Rapee et al., 2023) and poses a significant financial and personal burden (Pella et al., 2020). Compared to non-victimized youth, adolescents victimized by their peers are two to three times more likely to develop an anxiety disorder (Stapinski et al., 2014), and even isolated incidents of peer victimization can elicit feelings of worry and fear (Nishina, 2012). In turn, understanding the precise mechanisms through which peer victimization contributes to anxiety is critical for effectively identifying at-risk youth and preventing the development of anxiety disorders (Nikolić, 2020).

To better understand how peer victimization contributes to adolescents’ anxiety risk, it is useful to conceptualize it as a potential form of early-life adversity (i.e., stressful event causing serious emotional and/or physical harm; Jenkins et al., 2023) and draw from well-established conceptual frameworks that explain pathways from other forms of violence exposure to anxiety. Specifically, existing theoretical models of fear and safety learning propose that threat sensitivity is a key mechanism linking early-life adversity to anxiety development (Grasser & Jovanovic, 2021; McLaughlin et al., 2019). These models suggest that adversity-exposed individuals become hypervigilant to potential or ambiguous danger cues. Although adversity-induced hypervigilance can protect individuals in unsafe environments, maintaining a persistent state of alertness– especially in the absence of immediate danger – may contribute to chronic anxiety. Thus, threat sensitivity is theorized to elevate risk for anxiety and related disorders by increasing emotional reactivity and undermining fear discrimination (i.e., ability to distinguish between safe versus dangerous contexts) (McLaughlin et al., 2019; Teicher et al., 2016). While threat sensitivity has typically been conceptualized and studied as an individual differences variable, emerging evidence suggests that threat appraisals can also be dynamic, fluctuating within individuals based on context and social experience. For example, recent daily diary research demonstrates that the majority of variability in threat appraisals are attributable to within-person changes, as opposed to between-person differences, across one week (Mayer et al., 2022). Another study found that adolescents perceive greater threat on days that they witness interparental conflict, compared to days without interparental conflict, suggesting that situational factors can affect perceptions of threat (MacNeill & Fosco, 2022; Mayer et al., 2022)

Empirical findings support the theorized mechanistic role of threat sensitivity in links between peer victimization and anxiety, although such research has largely focused on adversity experienced in familial or neighborhood, rather than peer, contexts. For example, compared to non-abused children, children with a history of physical abuse exhibit heightened fear (i.e., perceived threat) in response to inter-adult anger (Hennessy et al., 1994). Similarly, adolescents more frequently experiencing or witnessing community violence (e.g., mugged; chased by gangs) report stronger threat appraisals in response to the recent violence exposure (Kliewer & Sullivan, 2008). Multiple studies link youth’s threat sensitivity, in turn, with subsequent anxiety risk. Not only do physically abused children show elevated attention to threatening cues, but such hypervigilance predicts greater anxiety symptoms (Pollak, 2008; Shackman et al., 2007). Heightened threat appraisals have similarly been documented as a mechanism linking community violence exposure to internalizing symptoms (Kliewer & Sullivan, 2008). Together these findings suggest that adversity experienced in familial and community settings increases risk for anxiety, in part via sensitizing youth to potential threats in their environments.

Whereas the aforementioned research has primarily focused on youth’s adverse experiences within their homes and neighborhoods, emerging theory conceptualizes peer victimization as a potential form of early-life adversity within the peer context (Jenkins et al., 2023). Additionally, we have proposed social threat sensitivity as a key mechanism linking peer victimization to the development of anxiety (Schacter et al., 2024). That is, adolescents who are repeatedly abused by peers are thought to become hypersensitive to potential (e.g., ambiguous) social threats in their environments, leading to persistently high levels of anxiety. There is some preliminary empirical support for a social threat sensitivity model of peer victimization and anxiety. In one cross-sectional study, peer victimization was positively associated with social threat sensitivity and anxiety symptoms among adolescent girls (Calleja & Rapee, 2020); girls who were more frequently bullied in the past month also reported stronger perceptions of threat in hypothetical ambiguous social situations and more severe anxiety symptoms. In another recent survey study (Schacter et al., 2024), social threat sensitivity mediated the concurrent association between peer victimization and anxiety symptoms, even after accounting for other forms of adversity (e.g., family maltreatment; community violence exposure). Specifically, adolescents who reported greater peer victimization in the past year experienced greater anxiety over the prior three months, and this association was partially explained by stronger perceptions of threat in hypothetical ambiguous social scenarios.

Despite consistent evidence for the long-term effects of peer victimization on anxiety symptoms (Christina et al., 2021) and initial empirical support for social threat sensitivity as a mediator of such links (Schacter et al., 2024), several gaps remain in our understanding. First, most research has relied on cross-sectional surveys, limiting conclusions about temporal sequencing. Additionally, studies that rely on one-time retrospective self-report measures of peer victimization can introduce recall bias (e.g., over- or under-estimation of victimization frequency) and mask meaningful within-person variability, especially insofar as peer victimization often ebbs and flows over time. Additionally, longitudinal studies that have demonstrated associations between peer victimization and anxiety have predominantly focused on how individual differences in peer victimization at one time predict differences in anxiety at a later time, typically months or years later. Yet, peer victimization can be quite unstable within individuals (Laninga-Wijnen et al., 2023), even changing from one day to the next (Nishina, 2012). Thus, it is important to consider intra-individual changes in peer victimization and their proximal psychological effects in adolescents’ daily lives; evidence of such short-term impact would suggest that even temporary, in addition to chronic, victims could benefit from intervention and support.

Daily diary assessment methods, wherein participants take brief surveys repeatedly over multiple days or weeks, offer a valuable tool for overcoming the limitations described above. By allowing adolescents to report their daily social experiences, thoughts, and emotions in real world contexts, these methods minimize retrospective bias, increase ecological validity, and capture realtime psychosocial functioning (Bolger et al., 2003; Russell & Gajos, 2020). Importantly, they extend understanding beyond individual differences (e.g., whether bullied youth experience greater anxiety than non-bullied youth) to isolate within-person processes (Modecki et al., 2019). For example, daily methods could elucidate whether adolescents exhibit greater social threat sensitivity and ensuing anxiety on days they are bullied compared to days they are not, using their typical daily experiences as a baseline for comparison. Additionally, by capturing repeated assessments within individuals over time, daily methods can shed light on discrete temporal processes, such as whether experiencing peer victimization on one day leads to heightened anxiety one or even two days later.

Although the question of whether peer victimization indirectly predicts anxiety via threat sensitivity has yet to be examined in a daily context, prior research provides evidence that peer victimization is linked with daily changes in emotion processing and mood among adolescents. For example, adolescents report increases in negative affect on days when they experience stressful peer events, such as conflict (Herres et al., 2016) and peer victimization (Laninga-Wijnen et al., 2024). Additionally, daily peer victimization has been linked to greater daily physical symptoms and overall psychological distress (i.e., aggregate of depressive and anxiety symptoms; Espinoza et al., 2013), However, little is known about daily associations between peer victimization and anxiety specifically, whether such associations may persist across multiple days, or the underlying daily mechanisms that explain proximal links between peer victimization and anxiety. To address these gaps, the current study implements a daily diary approach to examine how daily experiences of peer victimization contribute to state anxiety within and across days, and whether such links may be partially explained by aberrant threat processing.

### The present study

Although past research indicates that peer victimization increases adolescents’ risk for anxiety, the specific mechanisms underlying this relationship remain unclear, hindering progress towards evidence-based intervention and treatment approaches for peer victimized adolescents. Consequently, to better understand the microlevel processes that may contribute to the development and maintenance of anxiety symptoms among peer victimized youth, the current study leveraged a daily diary design. The study had two primary aims and hypotheses. First, we examined whether daily experiences of peer victimization predicted adolescents’ daily anxious affect and explored the duration of these associations. It was hypothesized that adolescents would exhibit increased anxiety on days when they experienced peer victimization compared to days without victimization, even after accounting for prior-day anxiety. Although we did not have specific hypotheses about the duration of these effects, we considered whether peer victimization on one day contributed to anxiety one or two days later. Second, we investigated whether daily increases in social threat sensitivity mediated the association between daily peer victimization and anxious affect, and we assessed the temporal span of these effects. It was hypothesized that daily experiences of peer victimization would be indirectly associated with elevated anxiety via daily increases in social threat sensitivity, while controlling for prior-day social threat sensitivity and anxiety. Similar to the first hypothesis, we did not have specific predictions about the duration of effects, although we minimally expected to detect same-day associations between peer victimization, threat sensitivity, and anxious affect. Given known gender differences in adolescent anxiety (Ohannessian et al., 2017) and the possibility of bidirectional effects, wherein anxiety elevates risk for future victimization (Christina et al., 2021), a series of exploratory sensitivity analyses considering gender moderation and reciprocal pathways from daily anxiety to peer victimization were also conducted.

## Method

### Overview of parent study

Data in the current study were drawn from Wave 6 of the Promoting Relationships and Identity Development in Education (PRIDE) Study, a multi-wave longitudinal study examining adolescents’ social-emotional development during high school. Participants in the PRIDE study were originally recruited as ninth grade students in November 2020 to participate in a five-wave online survey study spanning their ninth and 10^th^ grade school years. Participants were recruited from 38 high schools, the majority of which were public or charter schools located in urban or suburban areas. Over the course of one calendar year, participants completed five 20 – 30-minute online surveys every several months (Waves 1 – 5). As part of the Waves 1 – 3 surveys, participants also completed a brief online self-affirmation intervention designed to increase self-esteem and well-being. Specifically, participants were randomly assigned to one of three conditions where they completed a short writing exercise at the end of each survey (see Hoffman & Schacter, 2024 for additional details). Participants received a $10 e-gift card for each survey completed, and those who completed all five surveys were entered into a $100 e-gift card raffle. When participants were in 11^th^ grade in the spring of 2023, they were re-contacted and re-assented to participate in additional online surveys and a 2-week daily diary protocol (*n* = 195), described in greater detail below.

### Participants and procedure

The current study includes 195 participants (*M*_age_ = 16.48, *SD*_age_ = 0.53) who re-enrolled in the PRIDE study during 11^th^ grade and completed the Wave 6 daily diary procedures. Participants self-identified as 66% female, 27% male, and 11% non-binary, trans, not sure, or another gender and as 48% White, 20% Asian, 15% Black, and 17% another race/ethnicity. All participants provided online assent, and a waiver of parental consent was granted for the current study. For the daily diary protocol, participants responded to brief online surveys every day for 14 consecutive days. The surveys assessed daily experiences of peer victimization, threat sensitivity, and anxious affect, as well as several other constructs not examined in the current study (e.g., friendship experiences, school satisfaction). Daily surveys were sent to participants at 5:00 p.m. every day through Qualtrics SMS or email and closed for participation at midnight each night. Anyone who had not completed a daily survey by 9 p.m. each night received a reminder message. Participants were compensated for participation depending on their response rate to the daily surveys, with participants receiving a maximum of $30 if they completed all 14 daily surveys. The study’s procedures were approved by the Wayne State University Institutional Review Board.

Simulation studies suggest that multilevel models can yield unbiased estimates with a Level 2 sample size exceeding 50 and at least five within-person observations (McNeish & Stapleton, 2016). Additionally, prior daily diary studies with examining peer victimization with Level 2 sample sizes between 100 and 200 and Level 1 sample sizes between 5 and 14 have reliably detected significant within-person effects of daily peer victimization on adolescents’ daily adjustment, even in the context of relatively infrequent daily peer victimization experiences (e.g., Bakth et al., 2023; Espinoza, 2015; Nishina, 2012). Given our sample of 195 participants and up to 14 observations per participant (2,239 total data points), we expected sufficient power to detect the hypothesized effects.

### Daily compliance

The daily diary compliance rate was 82%, such that 2,239 of the possible 2,730 total daily surveys were completed. On average, participants completed 11.48 (out of 14) total surveys. The majority of participants (91%) completed at least five daily surveys, 80% completed at least 10 daily surveys, and 49% completed all 14 daily surveys.

### Measures

#### Peer victimization

Adolescents responded to six items each day assessing their peer victimization experiences. The items were modeled off of the cybervictimization and school victimization items used in a daily diary study among high school adolescents (Espinoza, 2015), originally derived from Espinoza et al., 2013 and Juvonen & Gross, 2008. Three items, which were only presented on school days, asked about experiences of school-based peer victimization (e.g., “Today a kid insulted or made fun of me.”). Three items, which were presented every day, asked about experiences of cybervictimization (e.g., “Today a kid spread rumors about me or excluded me online”). For each item, adolescents indicated whether it did (“Yes”) or did not (“No”) happen to them that day. From the six items, a dichotomous variable was created to indicate whether adolescents experienced any peer victimization (1) or no peer victimization (0) each day. If participants said “yes” to any singular item, they received a peer victimization score of 1 for that day.

#### Social threat sensitivity

To assess daily social threat sensitivity, a new assessment was created for this study. The items were adapted from existing measures of threat sensitivity thought to be appropriate for a daily context (Carver & White, 1994; Heffer & Willoughby, 2021). Specifically, adolescents responded to four items on a 5-point scale ranging from “Strongly disagree” (1) to “Strongly agree” (5). The four items were: “Today I felt worried that other kids were angry or annoyed with me,” “Today I was concerned that my friends didn’t like me,” “Today I worried that something bad would happen with my friends,” and “Today I was scared that I had upset a friend or classmate.” The four items were averaged to create mean daily social threat sensitivity scores for each participant. Multilevel confirmatory analyses for repeated measure data were used to calculate within- and between-person reliabilities (Geldhof et al., 2014). Alpha coefficients indicated good within-person (*α* = .83) and excellent between-person (*α* = .97) reliability. Additionally, to establish criterion validity of the novel measure, we examined whether social threat sensitivity (aggregated across the 14 days) was associated with related constructs available in the larger longitudinal dataset. Participants who reported higher average social threat sensitivity across the Wave 6 daily diary period also reported elevated “trait level” anxiety symptoms (*r* = .354, *p* < .001), somatic symptoms (*r* = .391, *p* < .001), and sleep disruptions (*r* = .348, *p* < .001) as measured on the Wave 6 baseline survey (Buysse et al., 1989; Spitzer et al., 2006; Walker & Garber, 2018). Average daily social threat sensitivity also prospectively predicted greater anxiety (*r* = .423, *p* < .001) and anticipatory processing of social situations (e.g., catastrophizing; Mills et al., 2013; *r* = .209, *p* = .012) one year later (Wave 7 survey). These associations provide initial support for the validity of the daily social threat sensitivity measure, as it relates consistently to theoretically linked constructs both concurrently and over time.

#### Anxious affect

Daily anxious affect was assessed via four items adapted from the Profile of Mood States for daily contexts (McNair et al., 1971; Yip et al., 2022). Adolescents were asked to think about their feelings and emotions throughout the day and rate how much they felt nervous, on edge, uneasy, and unable to concentrate on a 5-point scale ranging from “Very slightly or not at all” (1) to “Extremely” (5). The four items were averaged to create daily mean anxious affect scores for each participant. The measure exhibited satisfactory within-person (*α* = .75) and excellent between-person (*α* = .96) reliability.

### Control variables

Several control variables were included in analyses to account for potential confounds. At the within-person level, we controlled for the survey day (centered at day 1) and whether it was a school day (coded as 1) versus non-school day (coded as 0). To account for potential stability in social threat sensitivity and anxious affect from one day to the next, we also created lagged variables and controlled for auto-regressive effects of both variables. At the between-person level, we controlled for gender, race/ethnicity, and intervention condition using dummy coding. For gender and race/ethnicity, the reference group was determined based on the largest group size (female participants and White participants), and for intervention condition, the reference group was participants in the control condition. To account for individual differences in peer victimization across the two weeks, we also controlled for participants’ average peer victimization scores.

### Data analysis plan

Data analyses were conducted in Mplus Version 8 (Muthén & Muthén, 1998-2017) using multilevel modeling to account for nesting of repeated assessments within individuals. Before testing our main hypotheses, we estimated descriptive statistics, correlations, and intraclass correlations (ICCs) for the primary study variables. Intraclass correlations capture the proportion of variability attributable to between-person differences, as opposed to within-person changes. Given that participants completed an intervention during previous waves of the current study, a series of one-way Analysis of Variance (ANOVA) tests were also conducted to determine if the main study variables differed as a function of intervention condition.

To test our first hypothesis, we used multilevel modeling to examine the daily (within-person) relationship between peer victimization and anxious affect. The first model assessed same-day associations between peer victimization and anxious affect, while controlling for prior-day anxiety. The second and third models incorporated one-day and two-day lags to test cross-day effects. The lagged models evaluated whether peer victimization on one day predicted increased anxious affect the next day (one-day lag) or two days later (two-day lag), controlling for prior anxiety levels. In all models, continuous within-person predictors were person-mean centered.

To test our second hypothesis, we examined social threat sensitivity as a mediator of daily associations between peer victimization and anxious affect. Similarly, three models were tested. The first estimated same-day associations, testing whether daily peer victimization was associated with same-day increases in anxious affect indirectly via same-day social threat sensitivity, controlling for prior-day threat sensitivity and anxiety. The second model introduced a one-day lag to capture whether daily peer victimization predicted next-day threat sensitivity and anxious affect, and the third model used a two-day lag to examine a serial pathway where peer victimization one day predicted threat sensitivity the next day, which subsequently predicted anxious affect on the following day. Finally, we conducted a series of sensitivity analyses to determine whether daily associations were moderated by participant gender or bidirectional in nature.

Given the disadvantages of using traditional mediation analysis approaches with nested data, including the assumption of a normal distribution of the indirect effect, multilevel models were fit using the Bayes estimator using Mplus default settings with uninformative priors (Falk et al., 2024; Yuan & MacKinnon, 2009). All models were estimated using 1,000 iterations to achieve convergence. The Model Constraint command in Mplus was used to calculate indirect effects. Results are reported using 95% credible intervals (CIs) where CIs that do not include zero are considered statistically significant (Asparouhov & Muthén, 2010), and model convergence was evaluated using a Potential Scale Reduction value of <1.1.

## Results

### Descriptive statistics, correlations, and ICCs

Table 1 includes descriptive statistics and bivariate correlations for all primary study variables. On average, participants reported experiencing victimization on approximately one of the 14 days (*M* = .99, SD = 1.79). Additionally, 42% of participants (*n* = 82) reported at least one peer victimization incident over the two-week period. These rates align with findings from previous daily diary studies among comparable age groups (e.g., Nishina, 2012).

The ICC for peer victimization was 0.29, indicating that approximately one third of the variability in peer victimization was attributable to stable, between-person differences, whereas two thirds was attributable to daily fluctuations within individuals. The ICC for social threat sensitivity was 0.54, indicating that approximately half of the variability in social threat sensitivity was attributable to stable, between-person differences and half was attributable to daily fluctuations within individuals. The ICC for anxious affect was 0.61, indicating that 61% of the variability in anxious affect was attributable to stable, between-person differences whereas 39% was attributable to daily fluctuations within individuals.

A series of one-way ANOVAs tested whether there were mean differences in peer victimization, threat sensitivity, and anxiety across the 14 days as a function of intervention condition. There were no significant differences in number of days peer victimized, *F*(2, 192) = .307, *p* = .736, average daily threat sensitivity, *F*(2,192) = .715, *p* = .490, or average daily anxious affect, *F*(2, 192) = 1.619, *p* = .201.

### Same-day and lagged associations between peer victimization and anxious affect

As seen in Table 2, there was a significant within-person, same-day association between peer victimization and anxious affect, controlling for prior-day anxious affect and between-person differences in peer victimization. On days that adolescents were victimized by peers, compared to days they were not victimized by peers, they experienced increased anxious affect, over and above the effects of prior-day anxious affect. As seen in Table 3, there was also partial support for cross-day associations. Although peer victimization on one day did not predict increased anxious affect the next day, it did predict elevated anxious affect two days later.

In the same-day, one-day lag, and two-day lag models, there were also significant between-person effects of peer victimization on anxious affect. Adolescents who experienced greater average peer victimization across two weeks, compared to adolescents who experienced less average peer victimization, reported greater anxious affect across the two weeks, over and above the effects of gender, race, or intervention condition.

### Same-day and lagged indirect effects via social threat sensitivity

**Figure 1.**
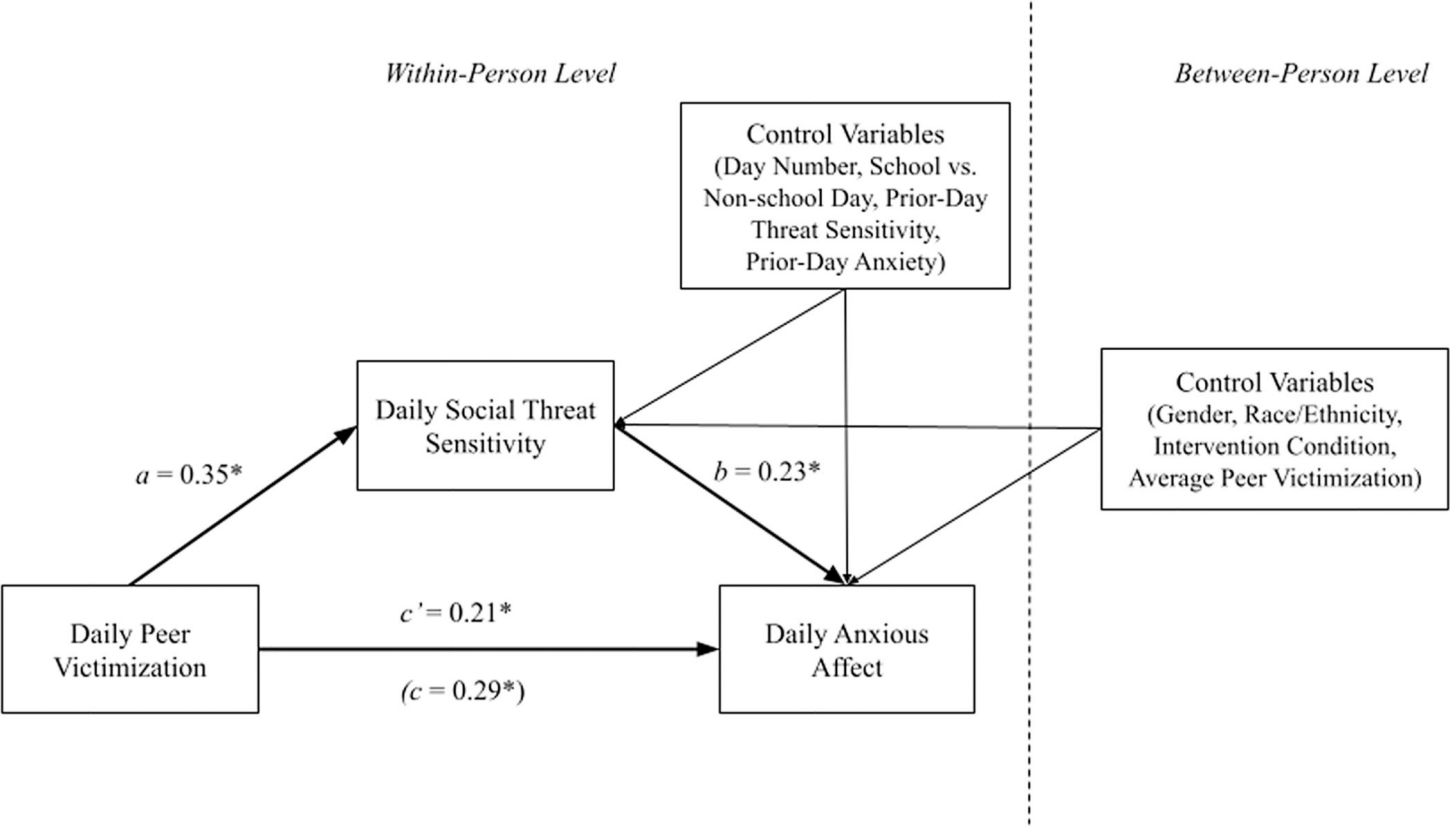
Daily social threat sensitivity as a mediator of the same-day association between peer victimization and anxious affect. *Note*. *Statistically significant effects, as indicated by a credible interval (CI) that does not include zero.

Results from the same-day mediation model indicated that the significant total effect of daily peer victimization on same-day anxious affect (*c* = 0.29, 95% CI = 0.18 – 0.42) was reduced, although still statistically significant (*c*’ = 0.21, 95% CI = 0.10 – 0.33), after accounting for daily threat sensitivity as a mediator (see Figure 1). There was also a significant within-person indirect effect of peer victimization on same-day anxious affect via social threat sensitivity (*ab* = 0.08, 95% CI = 0.05 – 0.11), providing evidence of partial mediation. Specifically, on days that adolescents were victimized by peers, they reported greater social threat sensitivity compared to days that they were not victimized by peers (*a*-path = .35, 95% CI = 0.22 – 0.47), controlling for prior-day social threat sensitivity. Greater daily social threat sensitivity, in turn, was associated with elevated same-day anxious affect (*b-*path = .23, 95% CI = 0.19 – 0.27), controlling for prior-day anxious affect. There was also a significant auto-regressive effect of social threat sensitivity from one day to the next (*b* = 0.08, 95% CI = 0.02, 0.13).

Although the one-day lag model indicated a non-significant effect of daily peer victimization on next-day anxious affect, we nevertheless considered the possibility that peer victimization on one day might predict increased anxious affect the next day indirectly via changes in social threat sensitivity. However, there was a non-significant indirect effect (*ab* = −0.03, 95% CI −0.07, 0.00). Daily peer victimization did not significantly predict next-day social threat sensitivity (*a*-path = −0.13, 95% CI = −0.26, 0.01), although social threat sensitivity was associated with same-day anxious affect (*b*-path = 0.25, 95% CI = 0.20, 0.29). There was also a significant auto-regressive effect of social threat sensitivity from one day to the next (*b* = 0.19, 95% CI = 0.14, 0.24).

Additionally, we examined a fully serial model to test whether daily peer victimization predicted increased anxious affect two days later via increases in next-day social threat sensitivity. However, there was a non-significant indirect effect (*ab* = 0.00, 95% CI −0.00 0.01). Daily peer victimization not did significantly predict next-day social threat sensitivity (*a-*path = 0.06, 95% CI = −0.07, 0.19), and next-day social threat sensitivity did not significantly predict anxious affect on the following day (*b*-path = 0.03, 95% CI −0.02, 0.08). There was also a significant auto-regressive effect of social threat sensitivity from one day to the next (*b* = 0.06, 95% CI = 0.01, 0.11). Thus, although social threat sensitivity partially mediated same-day associations between peer victimization and anxious affect, it did not explain lagged pathways.

### Sensitivity analyses

A series of exploratory sensitivity analyses were also conducted to assess the robustness of our main findings. Specifically, these analyses tested whether the documented associations between daily peer victimization, social threat sensitivity, and anxious affect varied by participant gender and whether daily associations were bidirectional in nature.

### Gender differences

To test for gender differences in same- and cross-day associations between peer victimization and anxious affect, we added cross-level interactions to the Aim 1 multilevel models. Given the small sample size of adolescents identifying as non-binary (*n* = 6), moderation analyses only compared associations across male-versus female- identifying participants. All results are tabled in Appendix A. For the same-day model (Table A1), the daily victimization X gender interaction was nonsignificant, indicating that peer victimization was related to same-day anxious affect for both boys and girls. For the one-day lagged model (Table A2), there was also nonsignificant gender moderation, such that daily peer victimization was unrelated to next-day anxious affect, regardless of participant gender. For the two-day lagged model, there was a significant daily peer victimization X gender interaction (see Table A2). Tests of simple slopes indicated that daily peer victimization was associated with anxious affect two days later for girls (*b* = .22, 95% CI = 0.08 – 0.37) but not boys (*b* = −.14, 95% CI = −0.39, 0.10).

To determine whether same-day or cross-day associations between peer victimization and social threat sensitivity varied by gender, we added daily peer victimization X gender cross-level interactions to the *a*-paths of the Aim 2 multilevel mediation models. None of the interaction terms were significant predictors of same- or cross-day social threat sensitivity, indicating that associations between daily peer victimization and social threat sensitivity were consistent across boys and girls (see Tables A3 and A4).

### Directionality

Although the main analyses were guided by a theoretical model wherein daily peer victimization precedes daily social threat sensitivity and anxious affect, additional same-day and cross-day models were tested to consider the possibility of bidirectional associations. In the first set of these models, we examined anxious affect as a same-day and cross-day predictor of peer victimization, controlling for prior-day victimization. As seen in Table B1, on days adolescents reported greater anxious affect, they reported increased peer victimization, over and above the effects of prior-day peer victimization. However, as seen in Table B2, there were no significant lagged effects of anxious affect on peer victimization one or two days later.

We also considered whether social threat sensitivity predicted changes in peer victimization. As seen in Table B3, on days adolescents reported greater social threat sensitivity, they also reported increased peer victimization, over and above the effects of prior-day peer victimization. However, as seen in Table B4, there were no significant lagged effects of social threat sensitivity on peer victimization one or two days later.

## Discussion

Leveraging a 14-day daily diary design, the current study offers new insights into pathways from peer victimization to adolescent anxiety. Our results demonstrate that even day-to-day peer victimization incidents can have proximal effects on adolescents’ anxious affect and offer novel evidence that daily associations between peer victimization and anxiety are partially explained by concurrent increases in social threat sensitivity. This is the first study, to our knowledge, that considers dynamic, fine-grained links between adolescent peer victimization and social threat sensitivity using repeated daily assessments. Moreover, the findings provide empirical support for emerging theories proposing that threat sensitivity partially accounts for the link between peer victimization and anxiety, offering new directions for prevention and intervention strategies among bullied youth.

The current study revealed several key findings. First, as hypothesized, adolescents reported greater anxious affect on days that they experienced peer victimization compared to days without victimization, even after controlling for prior-day anxiety. There was also partial support for spillover effects. Specifically, whereas daily peer victimization did not predict next-day anxiety, victimization on one day was linked with increased anxious affect two days later. Although previous daily diary studies have demonstrated short-term effects of peer victimization on specific features of anxiety, such as worry (Nishina, 2012), and broader indicators of psychological well-being, such as internalizing distress (Espinoza, 2015), our findings are among the first to isolate the nature and duration of the effects of peer victimization on daily anxious affect. These results suggest that even brief instances of victimization can elicit near-term feelings of nervousness and tension, independent of prior-day anxiety. The observed spillover effects further suggest that the psychological impact of daily peer victimization may unfold gradually over multiple days. Additionally, the use of a daily design allowed us to differentiate “state-like” patterns, reflecting within-person changes, from “trait-like” patterns, reflecting between-person differences. Although not a main focus of the study, we also found support for between-person associations, such that adolescents who experienced greater peer victimization across the two weeks also reported greater anxious affect. Thus, our findings build upon past research indicating that individual differences in peer victimization predict long-term anxiety risk (Christina et al., 2021) by highlighting how both between-person differences and temporary, within-person increases in peer victimization are related to anxious affect within and across days.

The most novel findings of the current study relate to the partial mediating role of threat sensitivity. Although theories linking early adverse experiences to anxiety implicate threat sensitivity as a developmentally relevant underlying mechanism (McLaughlin et al., 2019), research is only just beginning to consider how such models apply to peer victimization as a specific form of interpersonal adversity. Here, we found that same-day associations between peer victimization and anxious affect were partially mediated by daily threat sensitivity. On days when adolescents were victimized by peers, they exhibited heightened sensitivity to potential social threats, and this heightened threat sensitivity, in turn, was associated with greater anxious affect on the same day. Notably, these patterns held even after controlling for prior-day threat sensitivity and anxious affect. Thus, the present study suggests that daily elevations in threat sensitivity and anxious affect may be driven by corresponding daily experiences of peer victimization, rather than or in addition to reflecting a stable, trait-like tendency for vigilance and fear. These results offer empirical support for emerging theories that propose peer victimization as a potential form of interpersonal adversity (Jenkins et al., 2023) and threat sensitivity as one mechanism linking peer victimization to anxiety longitudinally (Schacter et al., 2024). Over time, repeated experiences of peer victimization could increase vigilance to possible social threats, potentially leading to the development of clinical anxiety disorders.

Despite evidence that social threat sensitivity partially mediated the same-day association between peer victimization and anxious affect, we did not find support for lagged mediational pathways. Although daily peer victimization predicted increased anxious affect two days later, this association was not mediated by changes in social threat sensitivity. One possible interpretation is that the links between victimization and threat processing may be more immediate, with social vigilance decreasing after adolescents recover by the next day. However, the two-day lagged effects of victimization on anxiety imply lingering emotional effects, potentially driven by mechanisms beyond threat sensitivity. For example, prior research among adolescents has identified daily coping strategies as mediators of the links between discrimination and well-being (Wang & Yip, 2020). Additionally, longitudinal studies have previously documented emotion dysregulation as a mechanism that explains prospective effects of peer victimization on internalizing symptoms (McLaughlin et al., 2009). In future research, it would be valuable to explore multiple mechanisms simultaneously and consider the timescale of their effects (e.g. immediate versus delayed) following victimization.

In the current study, exploratory sensitivity analyses further examined potential variation in patterns across gender and preliminarily considered bidirectional pathways between peer victimization, threat sensitivity, and anxiety. In terms of gender differences, tests of cross-level interactions indicated that daily associations between peer victimization, threat sensitivity, and anxious affect were generally consistent for boys and girls, with one exception. For girls, but not boys, peer victimization had lingering effects on anxiety symptoms two days later. One possibility is that this difference reflects girls’ heightened sensitivity to interpersonal rejection, insofar as past research provides evidence for amplified stress reactivity among girls (Burke et al., 2015; Vargas et al., 2021). Additionally, or alternatively, girls’ observed delays in recovering from daily peer victimization may be accounted for by other untested mechanisms, such as heightened rumination, that should be explored in future research.

Exploratory models testing alternative directional pathways further demonstrated that daily anxious affect was a significant same-day predictor of peer victimization, over and above the effects of prior-day victimization. That is, on days when adolescents felt more anxious than their typical anxiety levels, they were more likely to report being victimized peers. Prior research examining links between peer victimization and anxiety across multiple years has documented small but significant bidirectional associations, such that peer victimization not only elevates adolescents’ risks for future increases in anxiety, but adolescents presenting with elevated anxiety are more likely to become targets of victimization (Forbes et al., 2019). Together with the current findings, these results suggest that heightened worry and fear may manifest in behaviors (e.g. excessive reassurance-seeking; physical signs of nervousness) that increase adolescents’ vulnerability to peer mistreatment or lower their threshold for interpreting negative peer interactions as targeted victimization, potentially fueling a vicious daily cycle. Nevertheless, given that the current study examined bidirectional pathways across separate models, future work that accounts for reciprocal processes simultaneously will help further disentangle the temporal sequencing of peer victimization and anxiety.

### Limitations and future directions

The current results should also be understood within the context of several limitations. First, all assessments were based on adolescent self-reports. Although the daily diary design offers several methodological advantages, including minimization of retrospective bias and disentangling of within- and between-person processes, self-report bias may have inflated the observed associations between peer victimization, social threat sensitivity, and anxious affect. Future studies that incorporate additional reporting sources to assess peer victimization (e.g., peer nominations or teacher reports) could help strengthen the measurement. Furthermore, due to the relative infrequency of peer victimization incidents over the two-week period and the substantial non-normality of a continuous daily victimization variable, we used a dichotomous measure to indicate whether any victimization occurred on a given day. However, this binary approach does not capture the complexity of experiences for some youth who reported multiple types of victimization on the same day (e.g., being excluded and verbally harassed) or for adolescents who experience victimization less frequently (e.g., a few times a year). Additionally, the low prevalence of daily victimization, with only 42% of participants reporting any victimization over the two-week period, may have limited our power to detect within-person effects, especially given that the lagged modeling approach necessitated dropping data points on certain days. Future studies with larger sample sizes and more frequent assessments could consider how the duration and intensity of daily peer victimization experiences contribute to adolescents’ threat sensitivity and anxiety.

Second, although the study offered a novel contribution by examining threat sensitivity at the daily level, this approach necessitated the creation of a new daily assessment of social threat sensitivity. Existing self-report assessments of social threat sensitivity often involve participants reading and responding to hypothetical vignettes depicting ambiguous relational scenarios (e.g., Calleja & Rapee, 2020). However, lengthy daily questionnaires can increase participant burden and threaten data quality (Eisele et al., 2022). To maintain participant engagement over the two-week period, we prioritized brief assessments, as requiring participants to read and respond to vignettes for 14 consecutive days would likely have been overly burdensome. Although our measure demonstrated good within-person and excellent between-person reliability, as well as criterion validity, further validation of is warranted, and replication of the current findings using a multi-method approach would also be valuable. For example, pairing self-reports of peer victimization and anxious affect with physiological markers of threat sensitivity (e.g., startle reflex; Jovanovic et al., 2011) would offer a more rigorous test of our proposed mechanistic model.

Third, the majority of the current sample identified as female, and nearly half of participants identified as White. Although exploratory gender moderation analyses suggested that findings were largely consistent for boys and girls, we were underpowered to consider the potentially unique experience of youth identifying as non-binary, who are more likely to experience peer victimization (Huang et al., 2024) and mental health difficulties (Klinger et al., 2024) compared to their cisgender peers. We were also underpowered to test whether the current results varied (i.e., were moderated) by adolescents’ racial identity, although we did control for participants’ gender and race in all analyses. Thus, it would be important for future studies to replicate the current results with a more diverse sample to ensure generalizability.

## Conclusions

The current study demonstrated that daily experiences of peer victimization are associated with elevated anxious affect, within and across days, among adolescents. Additionally, findings implicated social threat sensitivity as one specific pathway through which peer victimization confers same-day risk for anxiety in adolescents’ daily lives. The findings underscore the emotional effects of even daily incidents of peer victimization, suggesting that parents, teachers, and school administrators should not minimize or dismiss adolescents’ infrequent experiences of being bullied as harmless. Additionally, intervention approaches that focus on understanding and modifying aberrant threat processing may offer one avenue of mitigating the distress of even temporary victims.

## Supplementary Material

1

**Supplementary material.** The supplementary material for this article can be found at https://doi.org/10.1017/S0954579425100394.

## Figures and Tables

**Table 1.** Within-person (daily) and between-person descriptive statistics and bivariate correlations

	*M*	*SD*	1.	2.
Between-Person (Level 2; *n* = 195)				
1. Average Peer Victimization	0.10	0.20	–	
2. Average Social Threat Sensitivity	2.11	0.75	0.352	–
3. Average Anxious Affect	2.06	0.82	0.228	0.591
Within-Person (Level 1; *n* = 2730)				
1. Daily Peer Victimization	0.08	0.27	–	
2. Daily Social Threat Sensitivity	2.06	0.95	0.227	–
3. Daily Anxious Affect	2.02	1.00	0.201	0.451

*Note*. All correlations significant at *p* < .001.

**Table 2.** Within-person (daily) and between-person same-day effects of peer victimization on anxious affect

	Outcome: Anxious Affect (*t*)	
Within-Person Predictors	Estimate	95% CI
Study Day	−0.01	−0.02, 0.00
School Day	0.13	0.06, 0.19*
Prior-Day Anxious Affect (*t*-1)	0.05	0.01, 0.10*
Daily Peer Victimization	0.30	0.17, 0.42*
Between-Person Predictors	Estimate	95% CI
Gender (*reference group = Female*)		
Male	−0.30	−0.55, −0.04*
Non-Binary	0.61	0.18, 1.06*
Race/Ethnicity (*reference group = White*)		
Asian	−0.03	−0.32, 0.27
Black	−0.20	−0.52, 0.12
Other	0.30	−0.02, 0.58
Intervention Condition (*reference group = Control*)		
Identity Affirmation	−0.01	−0.29, 0.25
Values Affirmation	0.07	−0.20, 0.36
Average Peer Victimization	1.76	0.94, 2.59*

*Note*.

* Statistically significant effect, as indicated by a credible interval (CI) that does not include zero. *t* refers to time, such that *t*-1 indicates a one-day lag.

**Table 3.** Within-person (daily) and between-person lagged effects of peer victimization on anxious affect

	Anxious Affect (*t* + 1)		Anxious Affect (*t* + 2)	
Within-Person Predictors	Estimate	95% CI	Estimate	95% CI
Study Day	−0.01	−0.02, −0.00*	−0.01	−0.01, 0.00
School Day	0.15	0.08, 0.21*	0.16	0.10, 0.22*
Anxious Affect	0.05	0.00, 0.10*	−0.03	−0.08, 0.02
Daily Peer Victimization	−0.03	−0.17, 0.10	0.13	0.00, 0.25*
Between-Person Predictors	Estimate	95% CI	Estimate	95% CI
Gender (*reference group* = *Female)*				
Male	−0.29	−0.53, −0.05*	−0.25	−0.51, −0.01
Non-Binary	0.62	0.14, 1.07*	0.62	0.15, 1.07*
Race/Ethnicity (*reference group* = *White)*				
Asian	−0.01	−0.32, 0.30	0.00	−0.30, 0.30
Black	−0.18	−0.52, 0.14	−0.19	−0.50, 0.11
Other	0.29	−0.03, 0.62	0.30	−0.02, 0.64
Intervention Condition (*reference group* = *Control)*				
Identity Affirmation	−0.02	−0.29, 0.27	−0.03	−0.32, 0.25
Values Affirmation	0.06	−0.21, 0.33	0.06	−0.22, 0.34
Average Peer Victimization	1.70	0.85, 2.53*	1.74	0.85, 2.66*

*Note*.

* Statistically significant effect, as indicated by a credible interval (CI) that does not include zero. *t* refers to time, such that *t* + 1 indicates anxiety one day later, and *t* + 2 indicates anxiety two days later.

## Data Availability

Data, code, and materials from the current study are available from the first author upon reasonable request.
