# Supplementary material for: Daily associations between peer victimization and anxious affect among adolescents: The role of social threat sensitivity
Source: Dev Psychopathol. Author manuscript; Available in PMC 2026 Feb 6. (PMC12459668; doi:10.1017/S0954579425100394)
Supplement: 1 [file NIHMS2095734-supplement-1.docx]

**Appendix A**

**Table A1.** Gender as a Moderator of the Same-Day Association Between Peer Victimization and Anxious Affect

|  | | | Outcome: Anxious Affect (*t*) | |
| --- | --- | --- | --- | --- |
| Within-Person Predictors | | | Estimate | 95% CI |
| Study Day | | | -0.01 | -0.02, 0.00* |
| School Day | | | 0.12 | 0.07, 0.18* |
| Prior-Day Anxious Affect (*t*-1) | | | 0.05 | 0.00, 0.10* |
| Peer Victimization | | | 0.30 | 0.15, 0.44* |
| Between-Person Predictors | | | Estimate | 95% CI |
| Gender (*reference group = Female)* | | |  |  |
| Male | | | -0.30 | -0.56, -0.05* |
| Non-Binary | | | 0.63 | 0.17, 1.06* |
| Race/Ethnicity (*reference group = White)* | | |  |  |
| Asian | | | -0.03 | -0.33, 0.30 |
| Black | | | -0.19 | -0.49, 0.11 |
| Other | | | 0.29 | -0.04, 0.61 |
| Intervention Condition (*reference group = Control)* | | |  |  |
| Identity Affirmation | | | -0.02 | -0.27, 0.26 |
| Values Affirmation | | | 0.06 | -0.20, 0.34 |
| Average Peer Victimization | | | 1.74 | 0.87, 2.58* |
| Cross-Level Interaction |  |  | Estimate | 95% CI |
| Daily Peer Victimization x Male | | | -0.02 | -0.30, 0.25 |

*Note*. *Statistically significant effect, as indicated by a credible interval (CI) that does not include zero. *t* refers to time, such that *t*-1 indicates a one-day lag.

**Table A2.** Gender as a Moderator of Cross-Day Associations Between Peer Victimization and Anxious Affect

|  | Anxious Affect (*t* + 1) | | Anxious Affect (*t*+2) | |
| --- | --- | --- | --- | --- |
| Within-Person Predictors | Estimate | 95% CI | Estimate | 95% CI |
| Study Day | -0.01 | -0.02, -0.00* | -0.00 | -0.01, 0.00 |
| School Day | 0.14 | 0.09, 0.21* | 0.17 | 0.11, 0.22* |
| Anxious Affect | 0.05 | 0.00, 0.10* | -0.03 | -0.08, 0.02 |
| Peer Victimization | 0.03 | -0.12, 0.17 | 0.22 | 0.08, 0.37* |
| Between-Person Predictors | Estimate | 95% CI | Estimate | 95% CI |
| Gender (*reference group = Female)* |  |  |  |  |
| Male | -0.27 | -0.54, -0.02* | -0.23 | -0.51, 0.04 |
| Non-Binary | 0.61 | 0.17, 1.07* | 0.60 | 0.17, 1.04* |
| Race/Ethnicity (*reference group = White)* |  |  |  |  |
| Asian | -0.02 | -0.32, 0.28 | 0.02 | -0.28, 0.33 |
| Black | -0.19 | -0.53, 0.13 | -0.19 | -0.53, 0.14 |
| Other | 0.28 | -0.02, 0.61 | 0.32 | -0.04, 0.62 |
| Intervention Condition (*reference group = Control)* |  |  |  |  |
| Identity Affirmation | -0.02 | -0.28, 0.27 | -0.02 | -0.31, 0.26 |
| Values Affirmation | 0.06 | -0.18, 0.33 | 0.07 | -0.21, 0.35 |
| Average Peer Victimization | 1.78 | 0.97, 2.57* | 1.85 | 0.91, 2.75* |
| Cross-Level Interaction | Estimate | 95% CI | Estimate | 95% CI |
| Daily Peer Victimization x Male | -0.22 | -0.50, 0.05 | -0.37 | -0.65, -0.09* |

*Note.* *Statistically significant effect, as indicated by a credible interval (CI) that does not include zero. *t* refers to time, such that *t*-1 indicates a one-day lag.

**Table A3.** Gender as a Moderator of the Same-Day Association Between Peer Victimization and Social Threat Sensitivity

|  | | | Outcome: Social Threat Sensitivity (*t*) | |
| --- | --- | --- | --- | --- |
| Within-Person Predictors | | | Estimate | 95% CI |
| Study Day | | | -0.01 | -0.02, -0.01* |
| School Day | | | 0.10 | 0.04, 0.16* |
| Prior-Day Social Threat Sensitivity (*t*-1) | | | 0.09 | 0.04, 0.14* |
| Peer Victimization | | | 0.38 | 0.23, 0.54* |
| Between-Person Predictors | | | Estimate | 95% CI |
| Gender (*reference group = Female)* | | |  |  |
| Male | | | -0.16 | -0.39, 0.07 |
| Non-Binary | | | 0.51 | 0.12, 0.90* |
| Race/Ethnicity (*reference group = White)* | | |  |  |
| Asian | | | -0.01 | -0.27, 0.25 |
| Black | | | -0.03 | -0.32, 0.24 |
| Other | | | 0.17 | -0.08, 0.46 |
| Intervention Condition (*reference group = Control)* | | |  |  |
| Identity Affirmation | | | 0.20 | -0.06 0.44 |
| Values Affirmation | | | 0.12 | -0.12, 0.36 |
| Average Peer Victimization | | | 2.02 | 1.30, 2.74* |
| Cross-Level Interaction |  |  | Estimate | 95% CI |
| Daily Peer Victimization x Male | | | -0.13 | -0.39, 0.17 |

*Note*. *Statistically significant effect, as indicated by a credible interval (CI) that does not include zero. *t* refers to time, such that *t*-1 indicates a one-day lag.

**Table A4.** Gender as a Moderator of the Cross-Day Association Between Peer Victimization and Social Threat Sensitivity

|  | Social Threat Sensitivity (*t* + 1) | | Social Threat Sensitivity (*t*+2) | |
| --- | --- | --- | --- | --- |
| Within-Person Predictors | Estimate | 95% CI | Estimate | 95% CI |
| Study Day | -0.01 | -0.01, 0.00 | -0.02 | -0.03, -0.01* |
| School Day | 0.14 | 0.08, 0.20* | -0.03 | -0.10, 0.04 |
| Social Threat Sensitivity | 0.20 | 0.16, 0.25* | 0.06 | 0.01, 0.12* |
| Peer Victimization | -0.14 | -0.27, 0.01 | 0.05 | -0.08, 0.17 |
| Between-Person Predictors | Estimate | 95% CI | Estimate | 95% CI |
| Gender (*reference group = Female)* |  |  |  |  |
| Male | -0.12 | -0.32, 0.08 | -0.19 | -0.45, 0.03 |
| Non-Binary | 0.43 | 0.11, 0.76* | 0.52 | 0.10, 0.88* |
| Race/Ethnicity (*reference group = White)* |  |  |  |  |
| Asian | 0.01 | -0.22, 0.24 | -0.05 | -0.33, 0.22 |
| Black | -0.04 | -0.27, 0.20 | -0.04 | -0.32, 0.22 |
| Other | 0.16 | -0.07, 0.40 | 0.16 | -0.12, 0.45 |
| Intervention Condition (*reference group = Control)* |  |  |  |  |
| Identity Affirmation | 0.16 | -0.06, 0.37 | 0.22 | -0.02, 0.44 |
| Values Affirmation | 0.09 | -0.11, 0.29 | 0.12 | -0.13, 0.34 |
| Average Peer Victimization | 1.64 | 0.97, 2.34* | 1.98 | 1.20, 2.79* |
| Cross-Level Interaction | Estimate | 95% CI | Estimate | 95% CI |
| Daily Peer Victimization x Male | 0.15 | -0.12, 0.43 | 0.03 | -0.26, 0.31 |

*Note.* *Statistically significant effect, as indicated by a credible interval (CI) that does not include zero. *t* refers to time, such that *t*-1 indicates a one-day lag.

**Appendix B**

**Table B1.** Same-Day Association Between Anxiety Symptoms and Peer Victimization

|  | Outcome: Peer Victimization (*t*) | |
| --- | --- | --- |
| Within-Person Predictors | Estimate | 95% CI |
| Study Day | -0.03 | -0.06, -0.01* |
| School Day | 0.78 | 0.53, 1.01* |
| Prior-Day Peer Victimization (*t*-1) | 0.45 | 0.07, 0.75* |
| Anxious Affect | 0.31 | 0.13, 0.46* |
| Between-Person Predictors | Estimate | 95% CI |
| Gender (*reference group = Female)* |  |  |
| Male | 0.31 | -0.08, 0.77 |
| Non-Binary | -0.15 | -0.82, 0.49 |
| Race/Ethnicity (*reference group = White)* |  |  |
| Asian | -0.06 | -0.53, 0.44 |
| Black | -0.02 | -0.47, 0.46 |
| Other | -0.41 | -1.00, 0.10 |
| Intervention Condition (*reference group = Control)* |  |  |
| Identity Affirmation | 0.09 | -0.32, 0.51 |
| Values Affirmation | -0.31 | -0.82, 0.09 |
| Average Anxiety | 0.47 | 0.24, 0.71* |

*Note*. *Statistically significant effect, as indicated by a credible interval (CI) that does not include zero. *t* refers to time, such that *t*-1 indicates a one-day lag.

**Table B2.** Cross-Day Associations Between Anxiety Symptoms and Peer Victimization

|  | Peer Victimization (*t* + 1) | | Peer Victimization (*t*+2) | |
| --- | --- | --- | --- | --- |
| Within-Person Predictors | Estimate | 95% CI | Estimate | 95% CI |
| Study Day | -0.04 | -0.08, -0.01* | -0.03 | -0.06, 0.00 |
| School Day | 0.92 | 0.55, 1.22* | 0.83 | 0.63, 1.16* |
| Peer Victimization | 0.39 | 0.05, 0.73* | 0.26 | -0.05, 0.59 |
| Anxious Affect | -0.05 | -0.21, 0.11 | 0.04 | -0.15, 0.21 |
| Between-Person Predictors | Estimate | 95% CI | Estimate | 95% CI |
| Gender (*reference group = Female)* |  |  |  |  |
| Male | 0.33 | -0.08, 0.76 | 0.38 | -0.09, 0.87 |
| Non-Binary | -0.26 | -0.99, 0.52 | -0.15 | -0.99, 0.69 |
| Race/Ethnicity (*reference group = White)* |  |  |  |  |
| Asian | -0.02 | -0.51, 0.46 | -0.14 | -0.68, 0.39 |
| Black | 0.01 | -0.53, 0.57 | 0.05 | -0.52, 0.60 |
| Other | -0.48 | -1.15, 0.08 | -0.60 | -1.30, 0.03 |
| Intervention Condition (*reference group = Control)* |  |  |  |  |
| Identity Affirmation | 0.12 | -0.31, 0.52 | 0.20 | -0.26, 0.71 |
| Values Affirmation | -0.36 | -0.82, 0.07 | -0.21 | -0.69, 0.28 |
| Average Anxious Affect | 0.57 | 0.33, 0.82* | 0.60 | 0.32, 0.87* |

*Note.* *Statistically significant effect, as indicated by a credible interval (CI) that does not include zero. *t* refers to time, such that *t*-1 indicates a one-day lag.

**Table B3.** Same-Day Association Between Social Threat Sensitivity and Peer Victimization

|  | Outcome: Peer Victimization (*t*) | |
| --- | --- | --- |
| Within-Person Predictors | Estimate | 95% CI |
| Study Day | -0.04 | -0.06, -0.01* |
| School Day | 0.74 | 0.47, 1.00* |
| Prior-Day Peer Victimization (*t*-1) | 0.36 | 0.09, 0.64* |
| Social Threat Sensitivity | 0.35 | 0.21, 0.52* |
| Between-Person Predictors | Estimate | 95% CI |
| Gender (*reference group = Female)* |  |  |
| Male | 0.31 | -0.08, 0.67 |
| Non-Binary | -0.20 | -0.85, 0.45 |
| Race/Ethnicity (*reference group = White)* |  |  |
| Asian | -0.10 | -0.54, 0.39 |
| Black | -0.16 | -0.76, 0.35 |
| Other | -0.41 | -0.93, 0.04 |
| Intervention Condition (*reference group = Control)* |  |  |
| Identity Affirmation | -0.04 | -0.45, 0.41 |
| Values Affirmation | -0.33 | -0.77, 0.04 |
| Average Social Threat Sensitivity | 0.63 | 0.36, 0.88* |

*Note*. *Statistically significant effect, as indicated by a credible interval (CI) that does not include zero. *t* refers to time, such that *t*-1 indicates a one-day lag.

**Table B4**. Cross-Day Associations Between Social Threat Sensitivity and Peer Victimization

|  | Peer Victimization (*t* + 1) | | Peer Victimization (*t*+2) | |
| --- | --- | --- | --- | --- |
| Within-Person Predictors | Estimate | 95% CI | Estimate | 95% CI |
| Study Day | -0.04 | -0.08, -0.02* | -0.03 | -0.06, -0.01* |
| School Day | 0.77 | 0.53, 1.04* | 0.78 | 0.52, 1.02* |
| Peer Victimization | 0.42 | 0.14, 0.73* | 0.26 | -0.10, 0.62 |
| Social Threat Sensitivity | -0.02 | -0.19, 0.14 | -0.05 | -0.23, 0.12 |
| Between-Person Predictors | Estimate | 95% CI | Estimate | 95% CI |
| Gender (*reference group = Female)* |  |  |  |  |
| Male | 0.22 | -0.17, 0.67 | 0.32 | -0.11, 0.70 |
| Non-Binary | -0.18 | -0.88, 0.43 | -0.15 | -0.95, 0.53 |
| Race/Ethnicity (*reference group = White)* |  |  |  |  |
| Asian | -0.05 | -0.54, 0.35 | -0.03 | -0.63, 0.47 |
| Black | -0.07 | -0.59, 0.41 | -0.09 | -0.60, 0.40 |
| Other | -0.36 | -0.85, 0.10 | -0.45 | -0.96, 0.10 |
| Intervention Condition (*reference group = Control)* |  |  |  |  |
| Identity Affirmation | -0.02 | -0.44, 0.38 | 0.02 | -0.37, 0.45 |
| Values Affirmation | -0.30 | -0.72, 0.11 | -0.31 | -0.74, 0.13 |
| Average Social Threat Sensitivity | 0.67 | 0.42, 0.96* | 0.70 | 0.43, 0.96* |

*Note.* *Statistically significant effect, as indicated by a credible interval (CI) that does not include zero. *t* refers to time, such that *t*-1 indicates a one-day lag.
